# The Health-Seeking Behavior of the Elderly with Non-Communicable Diseases in Coastal Areas of Vietnam

**DOI:** 10.3390/healthcare11040465

**Published:** 2023-02-06

**Authors:** Ho Minh Duy, Jakyoung Lee, Whiejong Han, Vasuki Rajaguru, Suk-Yong Jang

**Affiliations:** 1 Health Services of Thua Thien Hue, Hue City 530000, Vietnam; 2 Department of Global Health and Disease Control, Graduate School of Public Health, Yonsei University, Seoul 03722, Republic of Korea; 3 Department of Healthcare Management, Graduate School of Public Health, Yonsei University, Seoul 03722, Republic of Korea

**Keywords:** elderly, non-communicable diseases, health-seeking behavior, coastal

## Abstract

This study aimed to analyze the utilization of health care facilities and the health-seeking behavior of elderly people with non-communicable diseases and find the factors that affect them. A cross-sectional study was conducted in seven coastal areas of the Thua Thien Hue province, Vietnam, using a sample of 370 elderly people aged over 60 years. Chi-square and multiple logistic regression analyses were used to examine the factors associated with the utilization of health care services. The participants’ average age was 69.70 (SD), and 18% of them reported having ≥ two non-communicable diseases (NCDs). The results of the study showed that 69.8% of the total participants exhibited health-seeking behaviors. The findings also revealed that elderly people living alone, and those with an average or above-average income, had higher utilization of health care services. Participants with multiple NCDs exhibited more health-seeking behaviors than those with only one (OR: 9.24, 95% CI: 2.66–32.15, *p* = <0.001). The presence of health insurance and the need for health care counseling were also relevant ([OR: 4.16, 95% CI: 1.30–13.31, *p* = 0.016], [OR: 3.91, 95% CI: 2.04–7.49, *p* < 0.001], respectively). Health-seeking behavior is one of the most important positive implications for the aged population, as it encompasses one’s physical, mental, and psychological wellbeing. Future studies can aim at gaining an in-depth understanding of the same results, helping improve the health-seeking behavior of elderly people, and enhancing their quality of life.

## 1. Introduction

According to the World Health Organization’s forecast, the 21st century is an era of population aging; by 2050, approximately two billion of the world’s population will be over 60 years of age, up from 900 million in 2015. Of these people, 80% will be living in developing countries [1].

In Vietnam, over 11 million people are aged 60 and above, accounting for 11.8% of the country’s population. It is projected that by 2050, this number will increase to 29 million [2]. At this pace, Vietnam is becoming one of the few countries with the fastest aging population. Elderly people tend to suffer from more than one disease at a time, as all their organs start becoming functionally impaired. According to the Vietnam Ministry of Health, people over 60 years have 2.6 diseases, and those over 80 years have on average 6.8 diseases [3,4]. From 2009 to 2019, non-communicable diseases (NCDs) accounted for eight out of the top ten leading causes of death in Vietnam [5,6]. The rapidly increasing number of elderly people creates challenges for all countries, not just Vietnam. This significantly increases the burden of diseases on the health care system as well as the financing and management of NCDs in the community. Many countries are showing significant concern about this issue today and are looking for optimal remedies.

In coastal areas, the percentage of the population below the poverty line is higher than that of other areas; moreover, the national health criteria do not meet or fall below the country’s standard average. Other problems in these regions include access to hygienic water, access to hygienic latrines, and transportation difficulties. Therefore, in Decision No. 1559/QD-TTg, the Prime Minister and the government of Vietnam approved coastal areas to be designated extremely deprived areas [7]. 

Over the years, numerous efforts have been made to improve the provision of health care services to elderly people as well as their health management. National health insurance and primary health-care units were promoted and developed to provide free health insurance for elders 80 years of age and above, and vulnerable groups [8]. A health care project for elderly people was proposed for 2017–2025, with the goal of “The health care needs of the elderly to adapt to the aging population” [9]. The national strategy for 2015–2025 is to prevent and control NCDs [10].

Health-seeking behavior (HSB) is defined as “any action or inaction taken by individuals who perceive themselves to have a health problem or to be ill to find an appropriate remedy” [11,12]. HSB is measured in terms of the utilization of health care services, which is evidenced by the existence of multiple health-care services and the need for primary health care [13].

The HSB of elderly people is influenced by a variety of factors such as socio-economic status, age, gender, family, financial status, perceived health status and illness, type of illness, and access to services [14]. Ihaji et al.’s study reports that the decision-making processes of elderly people influenced their HSB. This study asserts that these decision-making processes are influenced by community norms and expectations, such as appropriate behavior for men and women in terms of social expectations, rights, power, access to resources for men and women, and health-related behavior, as well as education, gender, and regional organization [15]. Sarkisian et al.’s study states that elderly people having lower expectations about health as they aged was independently associated with their perception that HSB for age-associated conditions is “not very important.” In conclusion, these elderly people were uninformed of the possible benefits of seeking health care to address their age-related health issues [16].

However, even the increased provision of health care services for elderly people has failed to keep pace with the rapidly aging population, putting a great deal of pressure on building and implementing relevant policies, especially those related to health care, to help the elderly people in disadvantaged areas lead healthy lives. 

This study aimed to investigate the health-seeking behavior and associated factors elderly people with non-communicable diseases in the coastal areas of Vietnam.

## 2. Materials and Methods

### 2.1. Study Design

This study employed an observational, cross-sectional design to analyze the HSB of elderly people, as well as their utilization of medical facilities for NCDs at seven coastal areas in the Phu Vang district of the Thua Thien Hue province, Vietnam.

### 2.2. Population and Setting

This study was conducted in Phu Vang district, Thua Thien Hue province, Vietnam. The target areas were located in seven coastal areas. Initially, we used stratified random sampling based on the number of elderly people in each locality. The target population of this study was elderly people aged 60 years and over, having at least one NCD, and living in the coastal areas of Phu Vang district, Thua Thien Hue province, Vietnam.

### 2.3. Sample Size

The study participants aged over 60 years, living in the selected area for over 12 months and able to read and write the survey were included in this study. We used a formula to estimate the sample size [17,18,19], with *p*: the sample proportion, *p* = 0.62 [20]. Thus, we had a total of 370 subjects for the study. Areas with a larger number of elderly people were selected for the sample (Table 1). The formula for selecting elderly people was:

b =(a/ Total on. Eederly) × no. of selected

$$b=\frac{a}{7805}\;\times \;370\;(\mathrm{a: number of the elderly})$$

### 2.4. Variables and Measures

#### 2.4.1. Dependent Variables

The dependent variables considered in the study were HSB and utilization of medical facilities. Health-seeking behavior (HSB) was described as the health-related activities of elderly people with NCDs, including counseling, regular checkups, diagnosis, treatment, and follow-up. If the participants accessed any one of the activities, they were categorized as “yes”; if not, they were categorized as “no” [21,22]. Utilization of medical facilities was defined as the frequency of health-care facility visits and treatment history, and participants were categorized as either “yes” or “no”. 

#### 2.4.2. Independent Variables

The general characteristics of the participants were age group (60–69; 70–79; and 80 years and above) and gender (male and female). The religious categories considered were Buddhism, Christianity, and no religion. Levels of education considered were primary school or less and secondary school and above. Four categories of marital status were considered: married, single, separated/divorced, and widowed. The type of medical facilities were categorized as at home, at a primary health care unit, private clinic, district or provincial hospital or central hospital.

#### 2.4.3. Measures

A structured questionnaire was used as a tool for data collection. The questionnaire was prepared by the researcher based on the available information gathered from a review of the literature [20,21,22]. The structured questionnaire was used to collect information from the study participants under nine modules: (1) general information, (2) the history of non-communicable diseases and health-seeking behavior, (3) health-seeking behavior of elderly people with non-communicable diseases in the last 6 months. With regard to HSB and chronic diseases, the health-related data were self-reported. Participants were asked to rank their chronic diseases in order of importance. We chose the primary disease (i.e., primary diagnosis) based on the patient’s experience with diseases in order of importance. For example, if a patient had three chronic diseases, the highest-ranked disease was considered a primary disease condition in this study. The first part was comprised of about 12 questions. The second part consisted of 16 questions related to the history of non-communicable diseases and health status. The last part concerned health-seeking behavior. All the questions were open-ended (interview) and closed-ended questions that required a response of “yes” or “no” and multiple-choice questions.

Due to the pandemic, we were unable to conduct the content validity test; consequently, we analyzed the questionnaire’s reliability, and the Cronbach alpha score was 0.725. Participants received a response based on their disease diagnosis, experiences, symptoms of illness, and course of treatment. 

### 2.5. Data Collection Process

The pilot study was conducted in the month of September 2020, and it was initiated with face-to-face interviews of about 30 min by home visit. The investigator was provided with a list of participants with whom they had scheduled meetings at the participants’ homes. The study purpose and questionnaire were explained to the invited respondents, and the investigators obtained informed consent to confirm their volunteer participation. All the 370 people who were invited to participate agreed to be interviewed. The interviews began with oral consent and ended with signed confirmation. During the interview, the investigator explained the questions and asked the participants to fill in the answers in the data sheet. Finally, supervisors reviewed the collected data; among 370 participants, only 244 participants had HSB and were included in the final analysis.

The study was conducted under the supervision of the Research Ethics Council and approval of the Board of Directors of Health Services of Thua Thien Hue province (008-01-2020), Vietnam. Participants were asked to sign or provide fingerprints on the informed consent form after being informed of the freedom of withdrawal and other rights in a non-coercive environment. They were advised that the results would be used to propose and recommend the improvement to the population’s health and the health care system.

### 2.6. Data Analysis

HSB was calculated using Chi-square analysis or Fisher’s Exact test. Continuous variables were performed as means (standard deviation) and categorical variables were calculated as frequency (percentage). The associations of health care services with HSB and socio-demographic characteristics were examined using multiple logistic regressions with 95% confidence intervals (95% CI). All the data were analyzed by using SPSS 25.win (IBM corporation, Armonk, NY, USA) with a significant level of *p* < *0*.05 considered as statistically significant.

## 3. Results

### 3.1. Distribution of Demographic Characteristics and Health-Seeking Behavior of Elderly

The distribution of the demographic characteristics of the study participants is presented in Table 2. The mean age of the participants was 69.70 ± 6.6 (SD). The proportion between the two genders was approximately equal (49.2% male and 50.8% female). A majority of the participants were Buddhist (46.8%), 83.2% were married or cohabiting, and 91.4% lived with their relatives. Over half of the participants were engaged in fishery or agriculture (55.4%). Of the total number, 14.3% were under an average standard of economic status. Nearly two-thirds (58.4%) were located 3–5 km from the nearest primary health care unit. The proportion of participants with health insurance was 93.2%, and 54.9% of those had self-bought health insurance.

The most common disease in the study population was hypertension (42.4%), followed by musculoskeletal disease (17.8%). A total of 17.6% of patients had multiple NCDs, which is a high percentage. However, the percentage of participants who followed the treatment processes as per the prescription was just 81.3%. Of the total study population, 18.7% sought no treatment, bought medicines by themselves, or self-treated using traditional methods, and 15.9% felt that they had poor health status. Furthermore, 24.3% had complications with their diseases, and a third of a quarter of the participants required health care counseling (74.6%). Only 69.8% of the individuals used health care services for ongoing care and reported a recurrence of NCDs (86.8%). Mild illness (46.4%) and self-purchased medication or self-treatment using conventional means (53.6%) were the main causes for some participants’ failure to seek support from health care providers.

The results of analyzing the HSB of the study population who utilized health care services within the last six months are given in Table 1. A total of 370 elderly people had NCDs; of these, 224 exhibited HSB. Most of the participants utilized the medical facilities at PHCU (66.5%) due to its convenience of access from their houses (71.9%). More than half of the participants had national health insurance coverage (69.6%). Nearly half the participants (48.7%) agreed that the health care providers had excellent expertise for treating them.

The majority of the participants (75% of them) received outpatient care, and just 4% of them had to pay for the use of medical services. More than 90% of the participants expressed satisfaction with the medical services, including the equipment and medication. Despite the fact that 85.3% of participants followed up for a routine examination, 95.5% of participants felt that the treatment was acceptable. Over half the participants preferred obtaining treatment at a PHCU (50.9%), while 38.4% chose district hospitals.

### 3.2. Multivariate Logistic Regression Analysis of Factors Associated with Health-Seeking Behavior of Elderly People

Associated factors and HSB for utilization of medical facilities are given in Table 3. This shows that elderly people living alone exhibited 4.5 times less HSB than those who lived with their relatives (OR: 4.48, 95% CI: 1.016–19.78, *p* = 0.048). The results also indicated that economic status was related to HSB. People with average and above-average income seemed to have 2.8 times higher utilization of health care services than the poor and below-average group (OR: 2.81, 95% CI: 1.11–7.11, *p* = 0.029). Participants with multiple NCDs were likely to have nine times higher HSB than those with only one disease (OR: 9.24, 95% CI: 2.66–32.15, *p* = <0.001). Health insurance and health care counseling were also relevant factors that affected the HSB ([OR: 4.16, 95% CI: 1.30–13.31, *p* = 0.016], [OR: 3.91, 95% CI: 2.04–7.49, *p* < 0.001], respectively).

## 4. Discussion

The average life expectancy of both sexes of Vietnamese people is 75.8 years, which was consistent with the study results [22]. The mean age of the participants was 69.70. This mean age was similar to that reported by previous studies conducted in Vietnam and other countries [23,24,25]. In addition, it was reported in another study conducted in Thuy Chau ward, Huong Thuy town, Thua Thien Hue in three provinces in Vietnam [19], and a study conducted in rural areas of China [26]. However, this result was higher than the result of a study conducted in Bangladesh [27] and lower than the result of one conducted in Germany [28] and one conducted in Los Angeles (76.0 ± 6.9) [16]. This difference could be attributed to each country’s average life expectancy and the age distribution by groups. In developed countries, people aged 65 years and above are classified as elderly [29,30], while in Vietnam and many other developing and under-developed countries, people above 60 years of age are classified as elderly [31].

With respect to the presence of NCDs, this study found that hypertension was the most prevalent NCD among elderly people, followed by musculoskeletal disease, multiple NCDs, diabetes, and COPD. Very few cases of cancer were reported. The prevalence of NCDs among participants was much higher in this study than in previous reports, which reported higher hypertension, diabetes, COPD, and musculoskeletal diseases (arthritis) but a lower percentage of cancer, and in the rural Quoc-Oai district of Hanoi, Vietnam [23]; 6% diabetes, 0.7% cancer, and 8.3% musculoskeletal in Thuy Chau ward, Huong Thuy town, Thua Thien Hue province, Vietnam [19]; 32.3% hypertension, 4.9% COPD, and 3.2% cancer in Northwest Ethiopia [32]. However, this result was much lower than that reported by some previous studies: 62.15% hypertension among elderly ethnic minorities in Chiem Hoa district, Tuyen Quang province, Vietnam [20]; 40% multiple NCDs in southern provinces of Vietnam [24]; 56% hypertension, 64% diabetes in rural Bangladesh [27]; 47.8% hypertension, 34.8% musculoskeletal disease, 26.1% diabetes in Malaysia [33]; 56% hypertension, 21.8% diabetes in China [34]; 74% hypertension, 23% diabetes, 1% cancer in Albania [35]. This drop may be due to the national hypertension and diabetes screening program conducted in primary health-care units in recent years. This program helped to reduce unrecognized hypertension and diabetes diseases in the community [4,36]. However, due to limited infrastructure, the area was identified for increasing the accessibility to health care services in coastal areas, and for more screening for cancer and other NCDs [37]. In addition, the high consumption of salt and unhealthy lifestyles of people living in the coastal areas also affected the prevalence of NCDs in this area [38].

Out of the 224 participants who showed an HSB, 69.8% had been seeking health care for at least six months. The proportion of participants utilizing health care services in this study was much less than that reported by a previous study conducted in three regional areas in Vietnam. In the previous study, the proportion of HSB among elderly people in the North, Central, and South regions of Vietnam as well as the total were 87.39%, 96.24%, 86.86%, and 89.82%, respectively [39]; HSB was 83.3% at the Thua Thien Hue, Quang Tri, and Khanh Hoa provinces of Vietnam [39], and 83.7% in Assam, India [40]. The elderly in Vietnam made outpatient visits to health care centers an average of 4.3 ± 6.4 times per year [25]. This result was similar to the findings of previous studies conducted among the elderly population in Bangladesh, which was 33–67% depending on the disease [27], and higher than that reported in Pakistan at 43.3% [41] and the Dong Nai and Vinh Long provinces of Vietnam at 29.3% [24].

This study found that the type of family affected the utilization of health care services. The elderly living alone had 4.5 times less HSB than those who lived with their relatives. A study conducted in the rural Quoc-Oai District of Vietnam reported that elderly people living alone with chronic diseases was one of the main reasons for a shortening of life expectancy and an increase in emergency cases [23]. The movement of young laborers from rural to urban areas in search of work and the impact of socio-economic changes may be the reasons for elderly people living alone [42]. This result was similar to that of previous studies conducted in Vietnam and Nigeria but differs from that reported by a study conducted in Spain [25,43,44].

This study found that economic status and health insurance were two main factors that affected HSB. The elderly people falling in the poor and below-average income group seemed to have 2.8 times less utilization of health care services than above-average and average income groups. Economic conditions determine the willingness to pay for the costs related to HSB, such as participating in health insurance and paying a fee for health care services [45]. 

According to a study conducted in India, 81.2% of elderly people did not utilize health care services due to a lack of money [40]. In contrast, older adults who received financial support from their children had a positive relationship with the propensity to utilize health care services [38,46]. Some previous studies conducted in Vietnam, China, Ghana, and Nigeria also agreed that economic status was associated with seeking health care [42,43,45,47], but a study in India did not [40]. Furthermore, the presence of health insurance appeared to mitigate the impact of economic considerations, in addition to enhancing access to health care services [25,43,48,49]. Lack of health insurance may limit access to health care services, resulting in unmet needs and poor health outcomes among the aged, especially among the poor elderly [50]. In Vietnam, 14.3% of elderly people were living below the poverty line, and 6.8% of elderly people did not have health insurance. This proportion was higher than an average nation’s figures [7]. However, a study conducted in Vietnam by Nguyen and Giang showed that health insurance was not a predictor of access to health care services, which could be explained by the fact that health insurance services were limited in the studied areas [32].

Most previous studies reported that elderly people with higher numbers of NCDs were more likely to use health care services [25,48]. In contrast, a study conducted in Spain did not find any association [44]. In the current study, the proportion of multiple NCDs was high at 17.6% of the elderly. Elderly people with multiple NCDs had nine times higher HSB than those with only one significant disease. Furthermore, the study revealed that the demand for health care counseling is one factor that motivates the elderly to seek health care. Elderly people with a higher demand for health care counseling had 3.9 times higher HSB. A similar result was reported, which was that most elderly adults access health care services for counseling [51,52]. Therefore, policymakers and public health practitioners should consider elderly patient preferences regarding health care utilization in managing chronic diseases.

## 5. Conclusions

The purpose of this study was to improve the utilization of medical facilities among people aged over 60 years, improve HSB among older people with NCDs, and find out any associated factors. In the future, the government should pay more attention to this group by expanding the beneficiaries of health insurance support provided by the government, establishing nursing homes for elderly people, generating income for elderly people, improving health-care counseling services for elderly people through communication via social media, and encouraging people to change their unhealthy lifestyles, improve their health status, and prevent the incidence of NCDs. More research is required to understand HSB in older adults and utilize other physical and cognitive models to clarify the factors related to HSB. This clarification is crucial to understanding the HSB in older adults, as it can be the driving force behind assisting aging-in-place and providing essential references for decision-makers to generate context-specific incentive mechanisms and strategic plans in the future.

## 6. Limitations

This study had some limitations. First, this study used secondary data sources; the datasheets collected from the questionnaires did not mention the frequency of health-care facilities’ utilization directly related to the specific NCDs. Second, data collection was conducted in the third quarter of 2020, when the COVID-19 pandemic broke out; however, we had not assessed the impact of the pandemic on the HSB of elderly people. Most of the elderly people were dependent on others or family and had neither an income nor individual insurance; therefore, we did not include the economic status and health insurance correlation analysis. It should be considered in a further study. Finally, this study could not generalize the consistency of the findings due to the limited or small sample size. Vietnam is geographically diverse with various cultures, and therefore, there may be a variation in the HSB of people belonging to the various cultures. Furthermore, the health-care service delivery came from different regions. However, the results of this study could be compared to the results of other parts of Vietnam with similar features with respect to the socio-economic and cultural background in a future study.

## Figures and Tables

**Table 1:** Sampling estimate of study participants selected for each area.

No.	Area	No. Elderly (a)	No. Selected (b)
1	Phu Gia	1160	55
2	Vinh Ha	1215	58
3	Phu Xuan	1372	65
4	Vinh Xuan	948	45
5	Phu Dien	1587	75
6	Vinh An	974	46
7	Phu An	549	26
	Total	7805	370

**Table 2:** Health-seeking behavior of the study population who have utilized medical facilities within the last 6 months (*n* = 224).

Variables		Sample	
		N	%
Type of medical facilities	PHCU	149	66.5
	Private Clinic	4	1.8
	District/Provincial hospital	52	23.2
	Central hospital	19	8.5
Reason for using health facilities †			
Near house, convenience	No	63	28.1
	Yes	161	71.9
Excellent expertise	No	115	51.3
	Yes	109	48.7
Insurance cover	No	68	30.4
	Yes	156	69.6
Less waiting	No	107	47.8
	Yes	117	52.2
Type of treatment	Inpatient	28	12.5
	Outpatient	168	75.0
	Move to upper level	28	12.5
Payment for health care services	self	6	2.7
	Family members	3	1.3
	Insurance	215	96.0
Satisfaction of treatment	No	11	4.9
	Yes	213	95.1
Health facility satisfaction	No	19	8.5
	Yes	205	91.5
Effectiveness of treatment	No	10	4.5
	Yes	214	95.5
Regular checkup	No	33	14.7
	Yes	191	85.3
Preferred health care service †			
At home	No	168	75.0
	Yes	56	25.0
PHCU	No	110	49.1
	Yes	114	50.9
Private clinic	No	203	90.6
	Yes	21	9.4
District hospital	No	138	61.6
	Yes	86	38.4
Provincial/Central hospital	No	175	78.1
	Yes	49	21.9

PHCU = Primary health care unit; † Multiple response.

**Table 3:** Multiple logistic regression analysis of associated factors and health-seeking behavior for utilized medical facilities (n = 224).

Variables/Factors		Utilization of Medical Facilities (%)	OR	95% CI	*p*
Type of family	Alone	51.6	1.00		
	Not alone	71.7	4.48	1.01–19.78	0.048
Economic status	Under average	51.0	1.00		
	Average or upper	73.2	2.81	1.11–7.11	0.029
Health Insurance	No	27.3	1.00		
	Yes	72.9	4.16	1.30–13.31	0.016
Presence of non-communicable disease	One disease	64.0	1.00		
	Multiple NCDs	93.7	9.24	2.65–32.15	<0.001
Counseling service of NCDs	No	45.2	1.00		
	Yes	77.0	3.91	2.04–7.49	<0.001

NCDs = Non-communicable diseases; OR = Odds ratio; CI = Confidential interval.

## Data Availability

The data presented in this study are available on request from the corresponding author.
